# Molecular Evidence of Human Monkeypox Virus Infection, Sierra Leone

**DOI:** 10.3201/eid2506.180296

**Published:** 2019-06

**Authors:** Fei Ye, Jingdong Song, Li Zhao, Yi Zhang, Lianxu Xia, Lingwei Zhu, Idrissa Laybohr Kamara, Jiao Ren, Wenling Wang, Houwen Tian, Guizhen Wu, Wenjie Tan

**Affiliations:** China Center for Disease Control and Prevention, Beijing, China (F. Ye, J. Song, L. Zhao, Y. Zhang, J. Ren, W. Wang, H. Tian, G. Wu, W. Tan);; Sierra Leone–China Friendship Biological Safety Laboratory, Freetown, Sierra Leone (F. Ye, J. Song, Y. Zhang, L. Xia, L. Zhu, I.L. Kamara)

**Keywords:** monkeypox virus, viruses, Sierra Leone, sequence, molecular signature, zoonotic disease,

## Abstract

Monkeypox virus is a zoonotic disease endemic to Africa. In 2017, we confirmed a case of human monkeypox virus in Sierra Leone by molecular and serologic methods. Sequencing analysis indicated the virus belongs to the West African clade and data suggest it was likely transmitted by wild animals.

Monkeypox virus (MPXV), of the genus *Orthopoxvirus*, was identified in captive cynomolgus monkeys in Copenhagen in 1958 (*1*). The first documented case of human MPXV infection was reported in a patient from the Democratic Republic of the Congo in 1971 (*2*). Other outbreaks have occurred, including a large one in the United States in 2003, in which 47 confirmed and probable human cases of MPXV infection were identified after importation of wild rodents from Ghana (*3*). Phylogenetic analyses of MPXVs have revealed 2 distinct clades, West African and Congo Basin (*4*). In the past decade, human MPXV infections also have increased in Central and West Africa (*5*).

Ecologic niche modeling shows that Sierra Leone is in a geographic region suitable for transmission of MPXV (*6*). Several cases of human MPXV have been detected in West Africa, including a case in Sierra Leone in 1970 and another in March 2014 (*5*). We report confirmation of an MPXV infection in Sierra Leone in March 2017.

A 35-year-old man from Kpaku village, Galliness Perri chiefdom, Pujehun district, in southern Sierra Leone near the border with Liberia, sought treatment on March 16, 2017, for fever, body pain, malaise, dysphagia, and enlarged cervical lymph nodes. The patient reported hunting and eating squirrels ≈10 days before becoming ill, and traveling to Pelewahun gee bu in Bo district 3 days before his symptoms began. On March 17, he began having generalized vesicular skin eruptions. Clinicians sent vesicular swab specimens and blood samples collected on March 28 (12 days after the patient sought treatment) and on May 10 (day 55 after the patient sought treatment) to the Sierra Leone–China Friendship Biologic Safety Laboratory in Freetown, Sierra Leone. 

We conducted MPXV-specific real-time PCR, as previously described (*7*), and detected MPXV DNA in the day 12 blood sample (cycle threshold = 35.88). In addition, we performed MPXV-specific IgG ELISA, as previously described (*8*). The day 12 sample was positive at 1:800 dilution and the day 55 sample was positive at 1:3,200 dilution, demonstrating a >4-fold increase in the MPXV-specific antibody response during the patient’s recovery.

We amplified a 1,028-bp fragment of DNA for further Sanger sequencing (Figure, panel A) and submitted it to GenBank under Monkeypox virus Sierra Leone 2017 (accession no. MG906726). Phylogenetic analysis showed this strain is closer to MPXV isolates from the West African clade than from the Congo Basin clade (Figure, panel B). In addition, we found only a single nucleotide difference between the Sierra Leone 2017 fragment and MPXV Utrecht, UTC_1964 (Appendix Figure, panel A).

**Figure:**
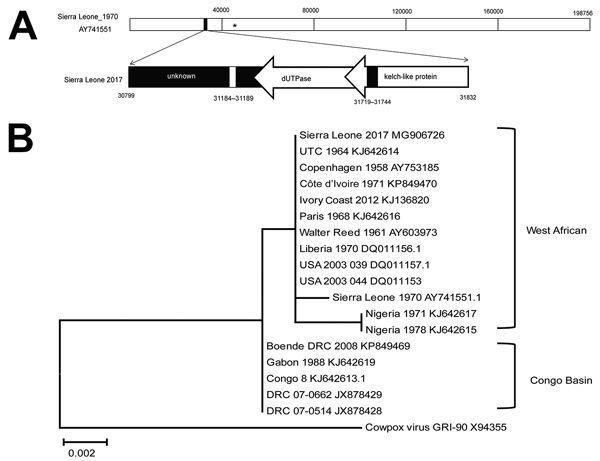
Phylogenetic analysis and molecular signatures of monkeypox virus (MPXV) Sierra Leone 2017 and other collected MPXV isolates. A) Schematic representation of the MPXV Sierra Leone 2017 genomic fragment by reference to genomic data on MPXV Sierra Leone 1970. MPXV Sierra Leone 2017 contains 3 parts: an unknown region, genes encoding dUTPase, and genes encoding partial kelch-like protein. *, binding position of primers used for real-time PCR detection. Bottom panel displays genes described. Arrows indicate direction of transcription. B) Phylogenetic relationships between genomic fragments of MPXV collected in Sierra Leone and other orthopoxviruses. Neighbor-joining phylograms constructed by using MEGA6 (https://www.megasoftware.net) and the maximum-likelihood method. Scale bar indicates nucleotide substitutions per site.

For further comparison, we selected previous MPXV sequences from NCBI (https://www.ncbi.nlm.nih.gov) and aligned these to Sierra Leone 2017 (Appendix Figure, panel B) using DNAMAN software (Lynnon Corporation, https://www.lynnon.com/dnaman.html). We found that Sierra Leone 2017 was closely related to UTC_1964. In addition, a 6-nucleotide deletion (GTATAC, a repeat unit) was more evident in the dUTPase region of Sierra Leone 2017 compared with other MPXVs from Africa. Also, a 25-nucleotide insertion (5× repeat of TCCAT unit) in the kelch-like protein-encoding region was more evident in the USA_2003_039 genome compared with most MPXV isolates from Africa. This insertion leads the N-terminal amino acids of the protein sequence that changed from EWNGMEWNGK in USA_2003_039 to VNNFEIK in Sierra Leone 2017.

Recent studies have shown that genome-region deletion in the Congo Basin MPXV clade can affect viral replication and pathogenicity (*9*). The deletion we noted in Sierra Leone 2017, which is absent in previously isolated MPXVs from Africa, might be a molecular signature and further evaluation is needed to determine whether it plays a role in viral replication and pathogenicity.

The case-patient lived in a region with large areas of tropical forest and many wild mammals. An ecologic niche model suggests this region is suitable for MPXV transmission (*6*). Considering the patient’s history of hunting and eating squirrels, and the lack of new cases among 16 identified community contacts in Pujehun, we hypothesize that the patient was likely infected by contact with wild animals. Of note, MPXV genetic evidence from this case-patient was closely related to UTC_1964, a strain that was isolated from an animal imported from Africa to the Rotterdam Zoo in 1966 (*10*). Because the reservoir species of MPXV remains unknown, we hypothesize that our case-patient was probably infected by exposure to wild animals and not from MPXV imported to Sierra Leone from Central Africa. However, further serologic surveys of animals in this region could provide useful evidence for the origin of MPXV strain Sierra Leone 2017.

In summary, we described a confirmed case of human MPXV in Sierra Leone in 2017 and molecular evidence hinting of its animal origin. We suggest MPXV circulation in wild animals and humans in West Africa requires more attention, and emphasize the value of local surveillance and molecular characterization of MPXV to help determine its origin.

Appendix Phylogenetic analysis and molecular signatures of monkeypox virus (MPXV) Sierra Leone 2017 and other collected MPXV isolates.
