# Supplementary material for: Molecular Evidence of Human Monkeypox Virus Infection, Sierra Leone
Source: Emerg Infect Dis. 2019 Jun;25(6):1220–2. doi: 10.3201/eid2506.180296 (PMC6537715; doi:10.3201/eid2506.180296)
Supplement: Appendix — Phylogenetic analysis and molecular signatures of monkeypox virus (MPXV) Sierra Leone 2017 and other collected MPXV isolates. [file 18-0296-Techapp-s1.pdf]

Article DOI: https://doi.org/10.3201/eid2506.180296

Molecular Evidence of Human Monkeypox Virus Infection, Sierra Leone

Appendix

Phylogenetic analysis and molecular signatures of monkeypox virus (MPXV) Sierra Leone 2017 and other collected MPXV isolates.

A

Isolate	Sierra Leone 2017	UTC_1964	Copenhagen 1958	Côte d'Ivoire 1971	Ivory Coast 2010	Paris_1968	Walter Reed 1961	Liberia 1970	USA 2003_039	USA 2003_044	Sierra Leone 1970	Nigeria 1971	Nigeria 1978	Boende DRC_2008	Gabon 1988	Congo_8	DRC 07-0662	DRC 07-0514
Sierra Leone 2017	—																	
UTC_1964	1	—																
Copenhagen_1958	16	16	—															
Cote d' Ivoire_1971	21	21	5	—														
Ivory coast_2012	6	7	10	15	—													
Paris_1968	12	11	5	10	6	—												
Walter_Reed_1961	16	16	0	5	10	5	—											
Liberia_1970	6	7	10	15	0	6	10	—										
USA_2003_039	31	31	15	10	25	20	15	25	—									
USA_2003_044	31	31	15	10	25	20	15	25	0	—								
Sierra Leone_1970	7	8	11	16	1	7	11	1	26	26	—							
Nigeria_1971	14	15	18	23	8	14	18	8	33	33	8	—						
Nigeria_1978	9	10	13	18	3	9	13	3	28	28	3	5	—					
Boende_DRC_2008	11	10	14	19	5	9	14	5	29	29	5	9	6	—				
Gabon_1988	11	10	14	19	5	9	14	5	29	29	5	9	6	0	—			
Congo_8	11	10	14	19	5	9	14	5	29	29	5	9	6	0	0	—		
DRC 07-0662	11	10	14	19	5	9	14	5	29	29	5	9	6	0	0	0	—	
DRC 07-0514	11	10	14	19	5	9	14	5	29	29	5	9	6	0	0	0	0	—

B

	30974	31164	31391	31585	31697
Sierra Leone 2017	TCTATACAAG	ATCTATATCATGTACAT-----------	TTTCC	CGATTGCCTAGTAGGC	TCCATTCCATTCCATTCCAT--CCCAT
UTC_1964	TCTATACAAG	ATCTATATCATGTACAT-----------	TTTCC	CGATTGCCTAGTAGGC	TCCATTCCATTCCATTCCAT--TCCAT
Copenhagen_1958	TCTATACAAG	ATCTATATCATGTACATGTACAT	TTTCC	CGATTGCCTAGTAGGC	TCCATTCCATTCCATTCCATTCCATTCCAT----------------------------CCCAT
Côte d'Ivoire_1971	TCTATACAAG	ATCTATATCATGTACATGTACAT	TTTCC	CGATTGCCTAGTAGGC	TCCATTCCATTCCATTCCATTCCATTCCATTCCAT---------------------CCCAT
Ivory Coast_2012	TCTATACAAG	ATCTATATCATGTACATGTACAT	TTTCC	CGATTGCCTAGTAGGC	TCCATTCCATTCCATTCCAT--CCCAT
Paris_1968	TCTATACAAG	ATCTATATCATGTACATGTACAT	TTTCC	CGATTGCCTAGTAGGC	TCCATTCCATTCCATTCCATTCCAT----------------------------------CCCAT
Walter_Reed_1961	TCTATACAAG	ATCTATATCATGTACATGTACAT	TTTCC	CGATTGCCTAGTAGGC	TCCATTCCATTCCATTCCATTCCATTCCAT----------------------------TCCAT
Liberia_1970	TCTATACAAG	ATCTATATCATGTACATGTACAT	TTTCC	CGATTGCCTAGTAGGC	TCCATTCCATTCCATTCCAT--CCCAT
USA_2003_039	TCTATACAAG	ATCTATATCATGTACATGTACAT	TTTCC	CGATTGCCTAGTAGGC	TCCATTCCATTCCATTCCATTCCATTCCATTCCATCCCATTCCATCCCAT
USA_2003_044	TCTATACAAG	ATCTATATCATGTACATGTACAT	TTTCC	CGATTGCCTAGTAGGC	TCCATTCCATTCCATTCCATTCCATTCCATTCCATCCCATTCCATCCCAT
Sierra Leone_1971	TCTATACAAG	ATCTATATCATGTACATGTACAT	TTTCC	TGATTGCCTAGTAGGC	TCCATTCCATTCCATTCCAT--CCCAT
Nigeria_1971	TCTATACAAG	CTCTATATCATGTACATGTACAT	CTTCC	CGATTGCCTAGTAGGT	TCCATTCCATTCCAT---CCCAT
Nigeria_1978	TCTATACAAG	CTCTATATCATGTACATGTACAT	CTTCC	CGATTGCCTAGTAGGT	TCCATTCCATCCCATCCCAT--TCCAT
Boende_DRC_2008	CCTATACAAG	CTCTATATCATGTACATGTACAT	TTTCC	CGATTGCCTAGTAGGC	TCCATTCCATCCCATCCCAT--TCCAT
Gabon_1988	CCTATACAAG	CTCTATATCATGTACATGTACAT	TTTCC	CGATTGCCTAGTAGGC	TCCATTCCATCCCATCCCAT--TCCAT
Congo_8	CCTATACAAG	CTCTATATCATGTACATGTACAT	TTTCC	CGATTGCCTAGTAGGC	TCCATTCCATCCCATCCCAT--TCCAT
DRC 07-0662	CCTATACAAG	CTCTATATCATGTACATGTACAT	TTTCC	CGATTGCCTAGTAGGC	TCCATTCCATCCCATCCCAT--TCCAT
DRC 07-0514	CCTATACAAG	CTCTATATCATGTACATGTACAT	TTTCC	CGATTGCCTAGTAGGC	TCCATTCCATCCCATCCCAT--TCCAT
					1 2 3 4 5 6 7 8 9 10

Appendix Figure. A) Nucleotide differences between Sierra Leone 2017 MPXV fragment and other MPXV isolates. Red indicates Congo Basin clade. B) Molecular signature of Sierra Leone 2017 MPXV fragments and other MPXV isolates. Numbers at top show genome positions of Sierra Leone 1970.

Underlines indicate the GTACAT repeat absent in Sierra Leone 2017 but present in Cophenhagen_1958 and other strains. Dotted lines indicate deletions. Red text indicates nucleotide differences between Sierra Leone 2017 MPXV fragment and other MPXV isolates. Numbers at bottom indicate the repeat times of T/CCCAT in MPXV genome.
